# Numerical simulation modeling and kinematic analysis onto double wedge-shaped airbag of nursing appliance

**DOI:** 10.1038/s41598-023-41619-y

**Published:** 2023-08-31

**Authors:** Yunxuan Xiao, Teng Liu, Chuizhou Meng, Zi’ang Jiao, Fanchao Meng, Shijie Guo

**Affiliations:** 1 Engineering Research Centre of the Ministry of Education for Intelligent Rehabilitation Devices and Testing Technology, Tianjin, 300401 China; 2 State Key Laboratory of Reliability and Intelligence of Electrical Equipment, Jointly Established By Hebei University of Technology and the Provincial Ministry, Tianjin, 300401 China; 3 School of Mechanical Engineering, Hebei University of Technology, Tianjin, 300401 China; 4 School of Electrical Engineering, Hebei University of Technology, Tianjin, 300130 China

**Keywords:** Biomedical engineering, Skin manifestations

## Abstract

In previous studies, the numerical modeling and analyzing methods onto industrial or vehicle airbags dynamics were revealed to have high accuracy regarding their actual dynamic properties, but there are scarcely airbag stiffness modeling and comfortableness investigations of nursing cushion or mattress airbags. This study constructs a numerical model illustrating the association between the stiffness property and the internal gas mass of the wedge-shaped airbag of nursing appliance, and then the airbag stiffness variation discipline is described based on various inflation volumes. To start with, based on an averaged pressure prerequisite, a dynamic simulation model of the wedge-shaped airbag is established by the fluid cavity approach. For this modeling, the elastic mechanical behaviors of airbag material are determined according to a material constitutive model built by the quasi-static uniaxial tensile test. Besides, verification experiments clarify that the presented modeling method is accurate for airbag stiffness behavior prediction, and then can be effectively applied into design and optimization phases of wedge-shaped airbags. Ultimately, based on the simulation and experimental results, it is found that the wedge-shaped airbag stiffness exhibits a three stages characteristic evolution with the gas mass increase. Then the mathematical relationship between the airbag stiffness and gas mass is obtained by numerical fitting, which provides a vital basis for structural optimization and differentiated control of nursing equipment airbags.

## Introduction

Incapacitated people or critically bedridden patients are prone to form pressure ulcers due to long-term compression of local tissues and blood circulation obstruction. In addition, severe pressure ulcer complications such as hypoalbuminemia, sepsis, and bone infection have even leaded to direct death for some patients^1–3^. Therefore, the prevention and treatment of pressure ulcers is an important part of daily care for these people. Traditionally, nursing staff regularly massages and rolls over as common measures to prevent pressure ulcers in patients^4–7^. However, these approaches possess inherent limitations, including a high demand for labor and an increased potential for infection transmission. Given these challenges, the research community has arrived at a consensus regarding the need to design a robotic solution capable of intelligently adjusting the sleeping position of patients using anti-pressure ulcer nursing equipment.

Addressing both comfort concerns and the prevention of pressure ulcers resulting from prolonged localized compression, an alternative approach involves the adjustment of airbag or waterbag height and stiffness to dynamically accommodate individual users. Notable instances of this method include the Body Perfect mattress in the United States^8,9^, which can real-time monitor a user’s physiological data, interpret their position, body contours, and intentions, and subsequently modify their posture. Additionally, the Leios airbag mattress^10^, a collaboration between the University of Tokyo and Molten Corp, integrates an internal tracking system to evaluate a user’s sleeping position comfort based on recorded daily sleep activities. The Leios mattress also offers basic temperature control and sleep position adjustment capabilities. Nonetheless, these mattresses often struggle to accurately regulate internal airbag or waterbag pressure and height, which limits their adaptability to a diverse range of users. To summarize, the aforementioned nursing equipment suffers from shortcomings such as insufficient quantitative control and limited intelligence, resulting in suboptimal suitability for patients with varying body types. Moreover, the intricate designs of existing products and redundancy within their control systems hinder their application in settings beyond hospital wards. Presently, a prevailing research direction for nursing cushions or mattresses revolves around the manipulation of arrays of airbags through differential internal pressure/height control. This technique seeks to achieve timed pressure distribution and personalized adaptation for users^11,12^. Consequently, researchers commonly employ numerical simulations to investigate the mechanical attributes of airbags utilized in nursing care products.

Drawing from the control volume method, Hu et al.^13^ formulated an airbag inflation control model to simulate the dynamic processes of airbag inflation and deflation. Li et al.^14^, on the other hand, employed CT scans to acquire the body geometry of male volunteers at the 50th percentile and subsequently developed finite element models of the back and hip in seated positions. Likewise, Chang et al.^15^ developed a model for substituting the human head, utilizing data from CT/MRI scans and experimentally obtained measurements. To characterize the elastic and hyperelastic properties of human tissue, they utilized the Ogden strain energy formula and the Proney series, respectively. Validation of their simulation model was achieved through static body pressure measurements, demonstrating its accuracy in capturing pressure distribution at the human-seat interface. In another study, Fongue et al.^16^ focused on modeling rubber airbags used in Rolling lobe air springs. They employed an exponential function to describe the amplitude correlation and hysteresis behavior of the airbag. Additionally, employing the Maxwell model, they accounted for the frequency dependence of the airbag and enhanced the air spring model to explore its damping effect. Nonetheless, the complexity of the model parameters and the lack of clear physical interpretations for many of these parameters posed difficulties for subsequent investigations into dynamic characteristics. Several scholars adopted diverse finite element modeling techniques and software platforms to assess the impact of load and temperature variations on the accuracy of hyperelastic material stiffness simulations. Cao et al.^17^ performed finite element simulations on the airbag under zero-stress, initial, and loaded states to analyze its static performance. Jeong et al.^18^, employing the Rebar, Shell, and Halpin–Tsai elements within Abaqus software, accounted for anisotropy and nonlinearity to construct a finite element model capturing the compression behavior of an air spring’s rubber airbag. A comparative analysis of spring deformation-force traits under varying cord angles through simulations and experiments indicated that the Rebar element yielded more accurate results across different cord angles. Furthermore, Li^19^ utilized Abaqus to assess and corroborate the stiffness characteristics of air springs in luxury cars, revealing the sensitivity of the air springs’ static stiffness to their effective area.

Despite the widespread attention and application of airbag nursing mattresses in pressure ulcer care, the stiffness characteristics and variations in loaded airbags remain unclear. While certain researchers have made notable strides in enhancing the simulation accuracy of hyperelastic material stiffness, these models face challenges in accurately capturing the stiffness dynamics of flexible airbags during inflation and deflation. Consequently, these models are unable to adequately depict the relationship between airbag pressure and its corresponding stiffness. To address this gap, the present paper undertakes a numerical modeling study of nursing airbags. It comprehensively investigates the load and compression displacement behaviors of airbags, with the goal of unraveling how airbag stiffness changes with differing gas masses. The ultimate objective is to achieve precise and adaptive control over the inflation process, thereby effectively catering to the distinct requirements of diverse nursing users.

## Materials and methodology

To address the aforementioned issues, this paper conducts an exploration into the numerical simulation modeling of airbag stiffness characteristics in the context of nursing devices. Additionally, it elucidates the correlation between external airbag loading and its compression displacement across varying internal gas masses. The initial step involves the introduction of double-wedge airbags employed within rehabilitation devices, alongside their typical application scenarios, presented within the “Materials and methodology” section. Given the team’s previous experience with rectangular airbags lacking rollover support, the shape of the wedge-shaped airbags is optimized and designed. Given the susceptibility of wedge-shaped airbags to inflation-related leakage, material airtightness becomes a pivotal consideration in material selection. The choice of material is influenced by the balance between effective sealing and maintaining the desired surface softness. Consequently, the decision is made to employ an 840D nylon composite thermoplastic polyurethane elastomer material for the airbag film, ensuring both appropriate sealing properties and surface texture.

**Figure 1:**
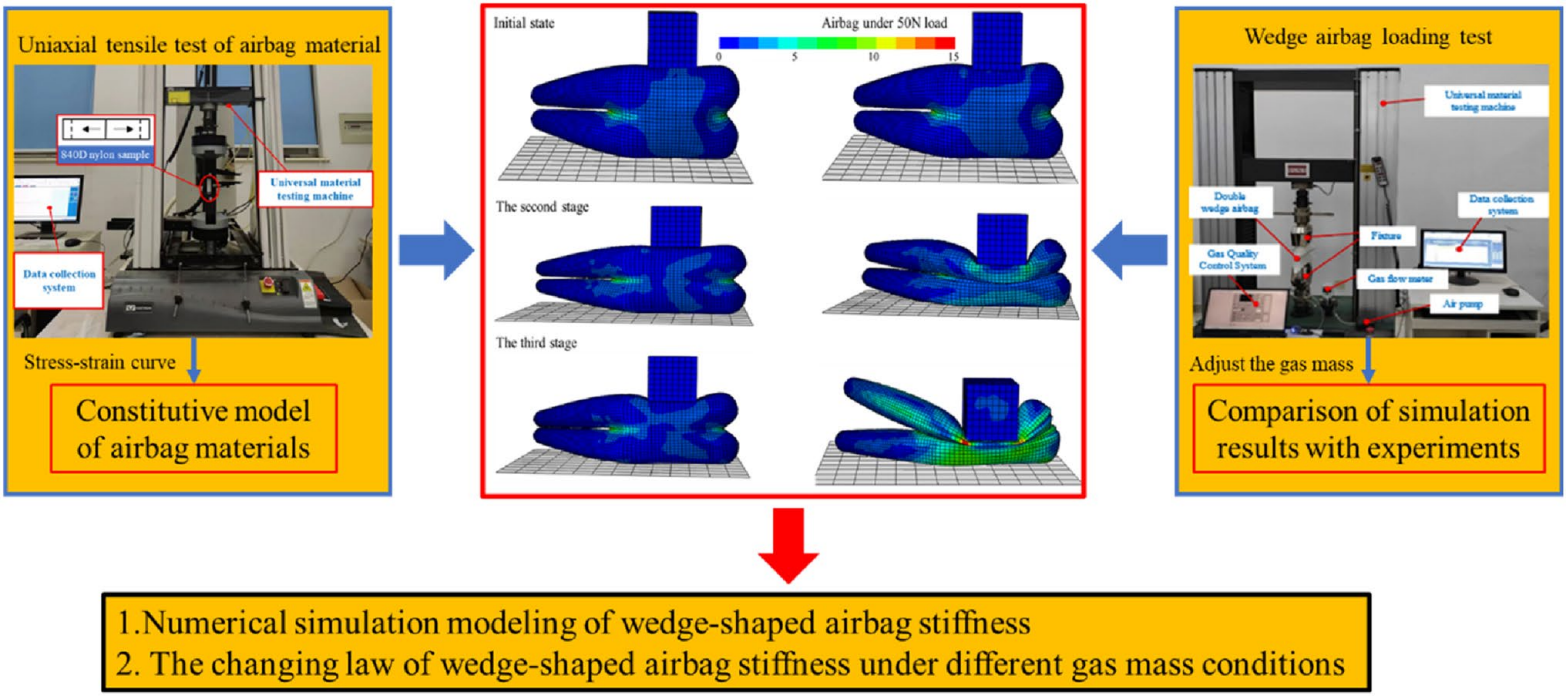
The research concept of the paper.

To establish a dependable simulation model for wedge-shaped airbag loading, this paper employs a universal material testing machine to conduct uniaxial tensile experiments, thereby gathering stress–strain data for the airbag film material. Based on simulations, the intrinsic model for the dual wedge-shaped airbag film material is determined, with the material parameters derived from the inherent structure of the airbag loading test. Subsequently, the paper establishes a simulation model for airbag stiffness under varying inflation volumes, utilizing the fluid cavity method where the air inside the airbag adheres to gas equation of state and gas exchange principles. Additionally, the paper designs load–displacement measurement experiments for individual airbags, the outcomes of which are used to verify the accuracy of the established model. Building on the simulation model above, this paper ultimately unveils the pattern of airbag stiffness alteration during the inflation process. The complete research concept is visually represented in Fig. 1.

### The double wedge-shaped airbag array unit

**Figure 2:**
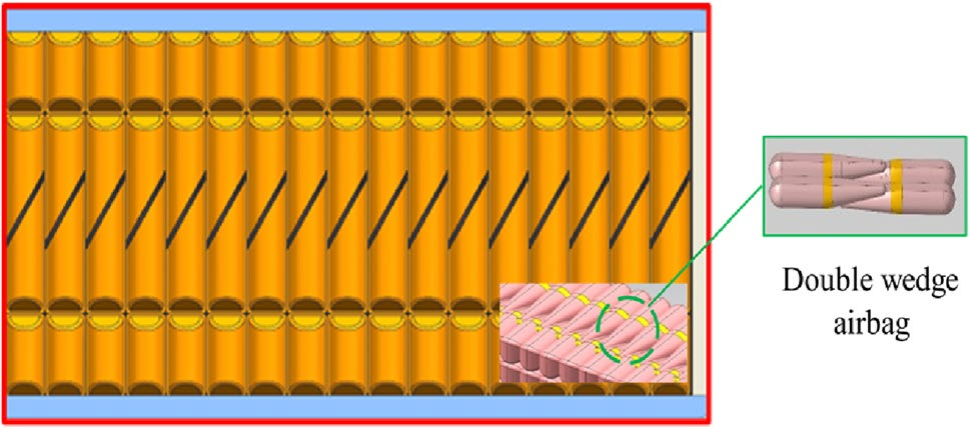
Wedge-shaped airbag array unit. Airbags in the array unit are coupled to each other.

Airbag nursing equipment usually has two forms: integral and airbag array combination, and the array airbag unit has the advantages of excellent control precision and comfort, which is the current mainstream design scheme. As the main force-bearing part of the nursing cushion or mattress, the single airbag structure design rationality in the array unit involves the comfort and safety of equipment above. So, the shape characteristics, curved surface and actual size must meet the requirements of the human body structure. The airbag is originally designed as a square single-layer structure, and based on the previous experimental data, the airbag array unit composed of the airbags can be used to make pressure ulcer care mattresses and pressure equalization cushions. However, the airbag above cannot achieve the proper inflation height and the rectangular airbag cannot provide sufficient support during the inflation and deflation process, so the airbag structure must be designed to be a double wedge-shaped air chamber that can be coupled with each other, which is illustrated in Fig. 2.

**Figure 3:**
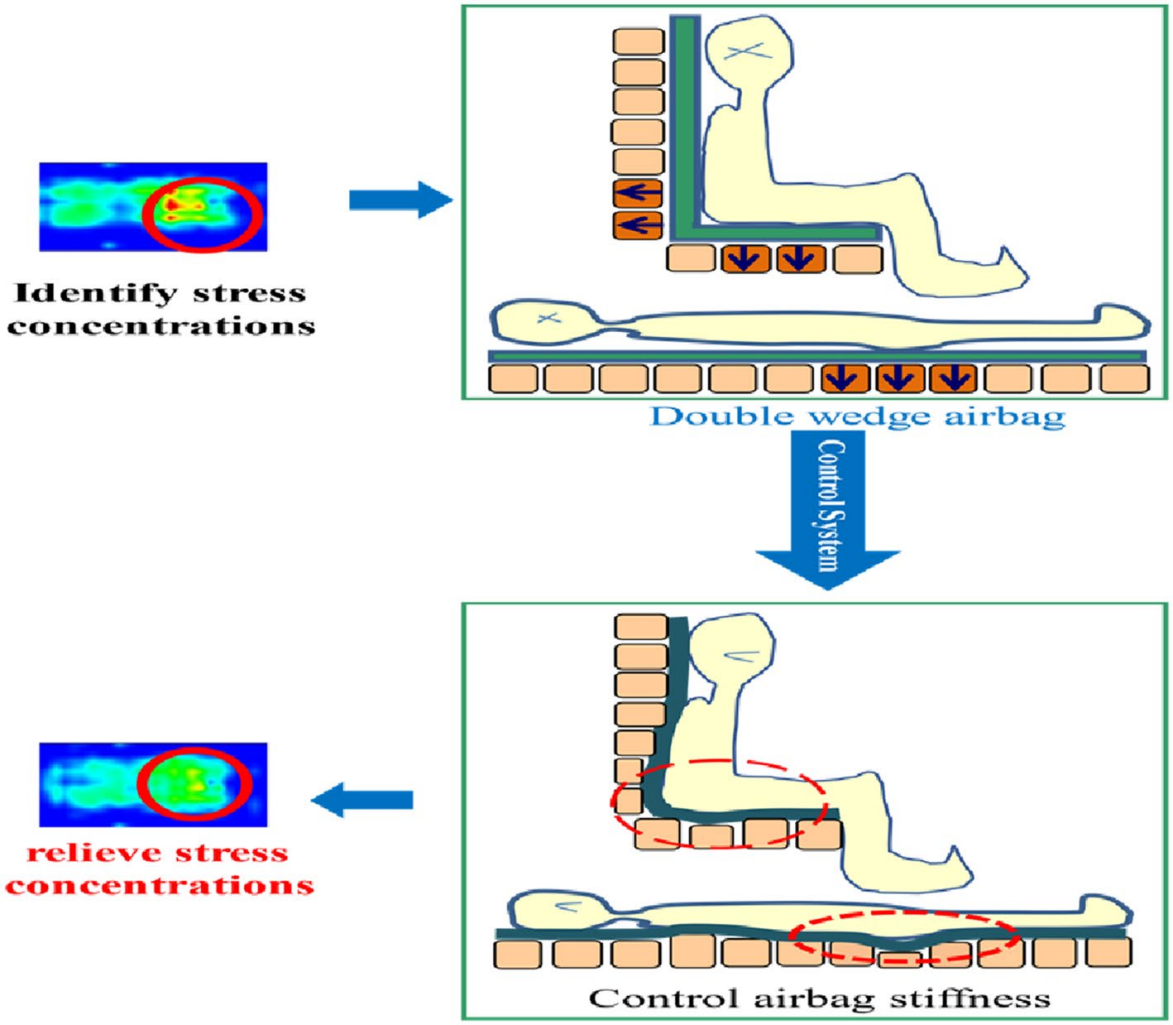
The working principle of airbag nursing mattress/cushion. Based on the pressure distribution, the pressure dispersion is realized by inflating and deflating the airbag.

The double wedge-shaped airbag, with dimensions measuring 350 mm along the longer side, 200 mm along the shorter side, and 100 mm in width, is manufactured from 840D nylon composite thermoplastic polyurethane elastomer (TPU) material using heat sealing techniques. The mechanical characteristics of the airbag are comprehensively detailed in Table 1. This airbag can be inflated to a maximum height of 150 mm through the introduction of air. As illustrated in the operational schematic presented in Fig. 3, the nursing apparatus operates under the control of an upper computer program, which orchestrates the activation and deactivation of the solenoid valve to facilitate the controlled inflation and deflation of the airbags. Importantly, each airbag is outfitted with an internal pressure sensor, thereby enabling real-time monitoring of the internal pressure. This integrated configuration provides the opportunity for customized adjustments in airbag stiffness and height, effectively accommodating diverse user postures and preferences.

**Table 1:** Data of the airbag mechanical properties.

	Material	Density [kg mm^−3^]	Long side [mm]	Short side [mm]	Width [mm]	Thickness [mm]
Values	840D nylon	1.15E-06	350	200	100	0.2

## Experimental

### Energy function of hyperelastic material

The mechanical behavior of the airbag film is different from that of general linear elastic materials, and the deformation process after being stressed is very complex, showing large displacement and large strain. So, in the airbag finite element analysis, the constitutive model of the airbag material (hyperelastic) plays a decisive role in the accuracy of the airbag simulation. For the incompressible or almost incompressible hyperelastic properties^20–22^, the tensile test is usually used to determine the model parameters. However, with various strains, it is still difficult to determine the constitutive model parameters. Therefore, based on the continuum theory, this paper conducts a finite element modeling on the polyurethane mechanical properties. The common polynomial strain energy function of hyperelastic material is as follows:

1 $$W=\sum_{i+j=1}^{N}{C}_{ij}{\left({I}_{1}-3\right)}^{i}{\left({I}_{2}-3\right)}^{j}+\sum_{i=1}^{N}\left[{1/D}_{i}{\left(J-1\right)}^{2i}\right],$$

2 $${I}_{1}={\lambda }_{v}^{2}+{2\lambda }_{v}^{-1},$$

3 $${I}_{2}={\lambda }_{v}^{-2}+{2\lambda }_{v},$$

4 $${I}_{3}={\lambda }_{1}^{2}{\lambda }_{2}^{2}{\lambda }_{3}^{2},$$

5 $$J={I}_{3}^{1/2},$$

where *W* is the strain energy, *N* is the Function class, *I*_1_, *I*_2_, and *I*_3_ are defined as the first-order, second-order, and third-order strain invariants, *J* is the volume ratio, *λ*_1_,* λ*_2_*, λ*_3_ are the main extension ratios, the *C*_*ij*_ is the material constant (MPa), usually obtained by experimental test, *D*_*i*_ is the material constant (MPa^-1^), which is related to the material compressibility.

### Airbag membrane quasi-static uniaxial tensile test

**Figure 4:**
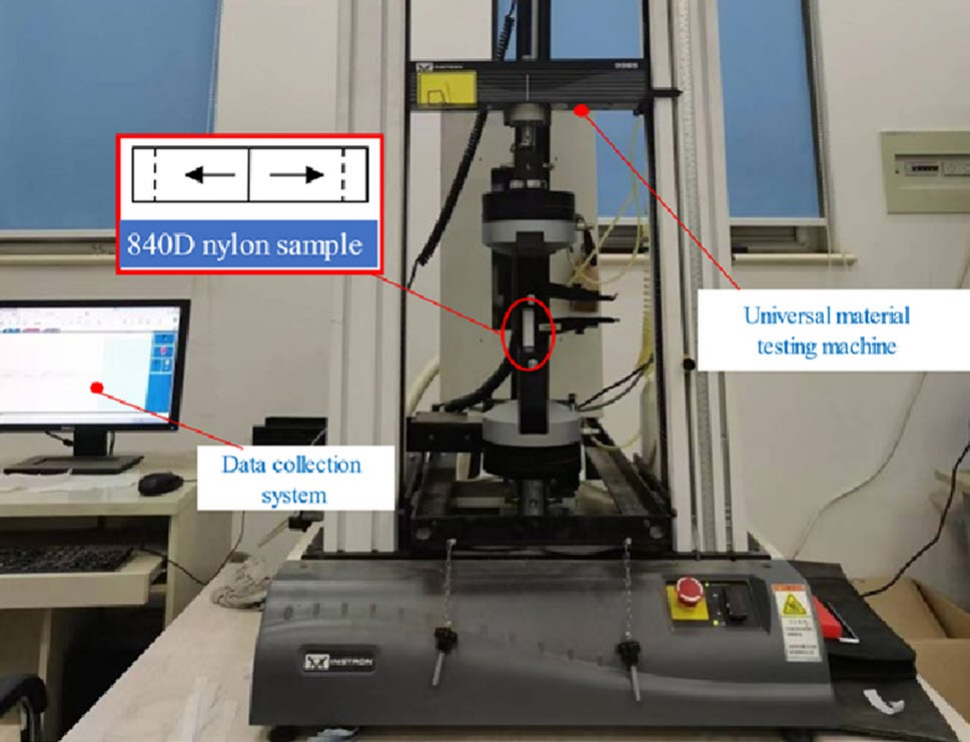
Material tensile test. The stress–strain curve of the airbag material is obtained by the universal material testing machine.

Eight identical double wedge-shaped airbag specimens made from the aforementioned material were employed for the tensile test. By calculating the average of their test results, an effort was made to mitigate potential experimental errors. Consequently, the physical characteristics of the airbag film were derived based on the quasi-static uniaxial tensile experimentation as described earlier. The uniaxial tensile testing of the airbag material was executed using an electronic universal material testing machine developed by Instron Corporation. Each of the eight specimens was individually affixed to the testing machine’s fixture, and subsequently, the specimen underwent controlled stretching in accordance with established testing standards (as illustrated in Fig. 4). To effectively negate the influence of viscoelasticity, the tensile test was conducted at a low rate: the lower clamp remained stationary, while the upper clamp vertically pulled the specimen upward at a controlled stretching rate of 100 mm/min. This procedure facilitated the acquisition of the specimen’s stress–strain curve. It is noteworthy that all eight specimens underwent the same testing conditions, with consistent tensile rates, to derive an averaged stress–strain curve.

**Figure 5:**
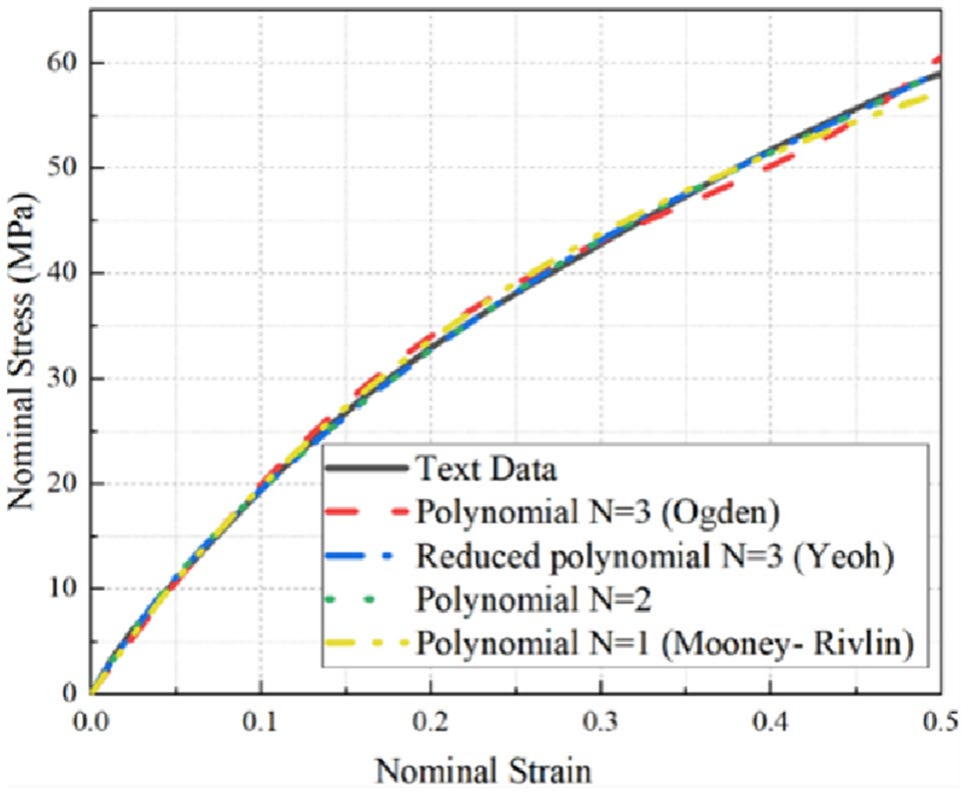
Comparison of simulation and experimental curves of models. Experimental data and simulation data of Ogden, Yeoh, Polynomial (N = 2), Mooney–Rivlin material constitutive model.

The simulation outcomes generated using all models were meticulously compared to the corresponding experimental data, as showcased in Fig. 5. Within this figure, the stress–strain relationship of the airbag film material, observed within the constraints of the experimental loading range, is represented by the solid black line. The remaining curves portray the results derived from commonly utilized material intrinsic fitting models in Abaqus, these being outcomes attained through simulations grounded in the experimental data. By undertaking a thorough analysis encompassing both the quantitative values and the discernible trends displayed by the experimental and simulated curves, the identification of the intrinsic model that most precisely characterizes the material properties of the airbag film becomes a feasible endeavor. Finally, the material parameters were determined according to the uniaxial tensile experiments exclusively. The Yeoh model in Abaqus is used for finite element simulation of double wedge-shaped airbag, and its parameters in Eq. (1) are fitted in ABAQUS. The airbag relevant simulation parameters are shown in Tables 2 and 3.

**Table 2:** Airbag material simulation parameters.

	C10	C20	C30
Values	31,587,917.0	− 765,825.66	− 3,065,876.18

**Table 3:** Gas initial parameter.

	Gas temperature [℃]	Molar mass [g/mol]	R [J/(kg·K)]	Initial pressure [MPa]
Values	23	29	8.314	0.1

When *j* = 0 and *N* = 3 in Eq. (1), the strain energy density function of Yeoh model is obtained:

6 $$W=\sum_{i=1}^{3}{C}_{i0}{\left({I}_{1}-3\right)}^{i}+\sum_{i=0}^{3}\left[{1/D}_{i}{\left(J-1\right)}^{2i}\right],$$

The material is assumed to be non-stretchable (*I*_3_ = 1) and isotropic^23,24^, meanwhile the material is always in a uniaxial tensile state during working process. According to Eq. (5), the simplified function can be obtained:

7 $$W=\sum_{i=1}^{3}{C}_{i0}{\left({I}_{1}-3\right)}^{i}.$$

The airbag material being assumed to be incompressible, the relationship between the strain potential energy and the engineering stress can be obtained experimentally in the uniaxial stretching process:

8 $${\sigma }_{i}=\frac{\partial U}{\partial {\lambda }_{v}}=\frac{\partial U}{\partial {I}_{1}}\frac{\partial {I}_{1}}{\partial {\lambda }_{v}}+\frac{\partial U}{\partial {I}_{2}}\frac{\partial {I}_{2}}{\partial {\lambda }_{v}},$$

where *σ*_*i*_ is the engineering stress (MPa), *λ*_*υ*_ is the uniaxial stretching ratio measured by the experimental method. Strain invariants can be obtained.

### Governing equations of airbag stiffness properties simulation

The control volume method based on the pressure equalization assumption and the fluid–structure interaction (FSI) method are common air bag modeling methods. But when the inflatable air bag is loaded, the gas inside the airbag can be expanded and has compressibility. When the airbag is loaded, the airbag will deform. If the force is removed, it will return to its original state. Therefore, the airbag internal gas physical properties play a decisive role in the mechanical properties analysis. The airbag load force transmission depends on its deformation, so there is a huge error in the analysis result that the internal pressure is equivalent to the uniform pressure.

The fluid cavity method in ABAQUS is a special fluid–structure interaction modeling method for airbag inflation simulation.

For the transient simulation, the airbag inflation is regarded as an adiabatic process, moreover, the airbag internal temperature remains uniform at any moment. The values of gas internal pressure, gas constant and temperature are set in simulation for describing the injected gas. Since the charged gas volume follows the ideal gas equation, its real time volume can be calculated by the following equation:

9 $$\overline{V }=\overline{V }\left(p,\theta ,m\right),$$

10 $$\frac{d\overline{V}}{dp }=-\frac{m}{{\rho }_{R}K},$$

where $\overline{V }$ (L) is the gas volume inside the airbag, *p* is the pressure (MPa), $\theta$ is the gas temperature (K), *ρ*_*R*_ is the reference fluid density, *K* is the fluid bulk modulus, and *m* is the gas mass (kg). With the airbag gas being assumed to be an ideal gas (its heat capacity remains constant), its specific thermodynamic energy can be determined by the airbag thermodynamic energy at any time:

11 $$E=\frac{m}{M}{C}_{v,m}\theta ,$$

12 $$e=\frac{E}{\rho 0},$$

where *E* is the thermodynamic energy (J), *M* is the gas molar mass, *C*_*v,m*_ is the molar heat capacity at the constant volume (J·mol/K), *e* is the specific thermodynamic energy, and *ρ* is the density (kg/m). Furthermore, the gas mass and density in the airbag has the following relationship:

13 $$m=\sum_{e}{m}^{e},$$

14 $$\rho \left(p,\theta \right)=\frac{p+{p}_{A}}{R\left(\theta -{\theta }_{A}\right)},$$

where *R* is the gas constant, *p*_*A*_ is the initial pressure (MPa), ${\theta }_{A}$ is the initial temperature (K). The airbag gas volume inside the airbag under adiabatic conditions meets the following relationship:

15 $$\overline{V }\left(p,\theta \right)=\sum_{e}{\overline{V} }^{e}\left(p,\theta \right)=\frac{\sum_{e}{m}^{e}}{\rho (p,\theta )}=\frac{m}{\rho (p,\theta )},$$

16 $$\frac{d\overline{V}}{dp }=-\frac{m}{{\rho }^{2}}\frac{d\rho }{dp}=-\frac{mR\left(\theta -{\theta }_{A}\right)}{({p+{p}_{A})}^{2}},$$

17 $$\Delta \overline{V }\left(p,\theta \right)=\frac{\Delta m}{\rho (p,\theta )}.$$

### Finite element simulation modeling of airbag stiffness properties under various gas mass

**Figure 6:**
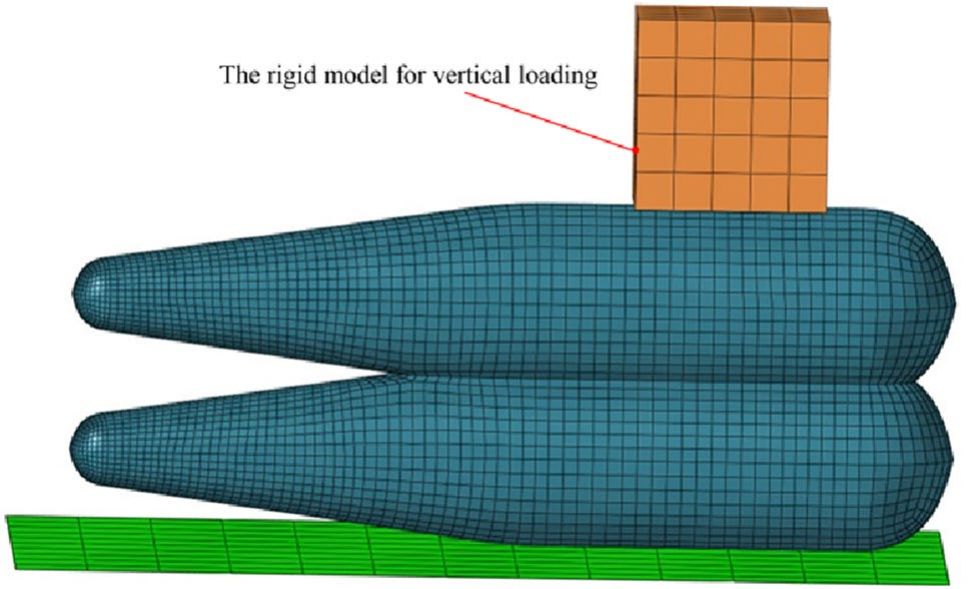
Airbag finite element model with vertical loading. The floor and load are non-discrete rigid bodies, and the airbag model is the hyperelastic film.

Due to the pliable nature of the airbag material and the significant deformation experienced during the loading process, the double-wedge airbag is divided into a larger number of mesh elements. Conversely, the loading block of the universal material testing machine maintains its shape during contact with the airbag. Reducing the quantity of mesh elements for the loading block can enhance computational efficiency. However, to ensure the precision of the simulation model and prevent excessively disparate mesh sizes on the contact surfaces, it is essential to establish an appropriate mesh size for the loading block. As illustrated in Fig. 6, the elastic double-wedge safety airbag with an intermediate connection is discretized into 6082 linear square membrane elements with a side length of 5 mm. Within the finite element simulation, both the bed board and the load applied by the human body are assumed to be rigid. Consequently, a rigid body model comprising 150 shell elements with a side length of 10 mm is generated at the top of the safety airbag. This allows for the implementation of vertical loading in the simulation.

**Figure 7:**
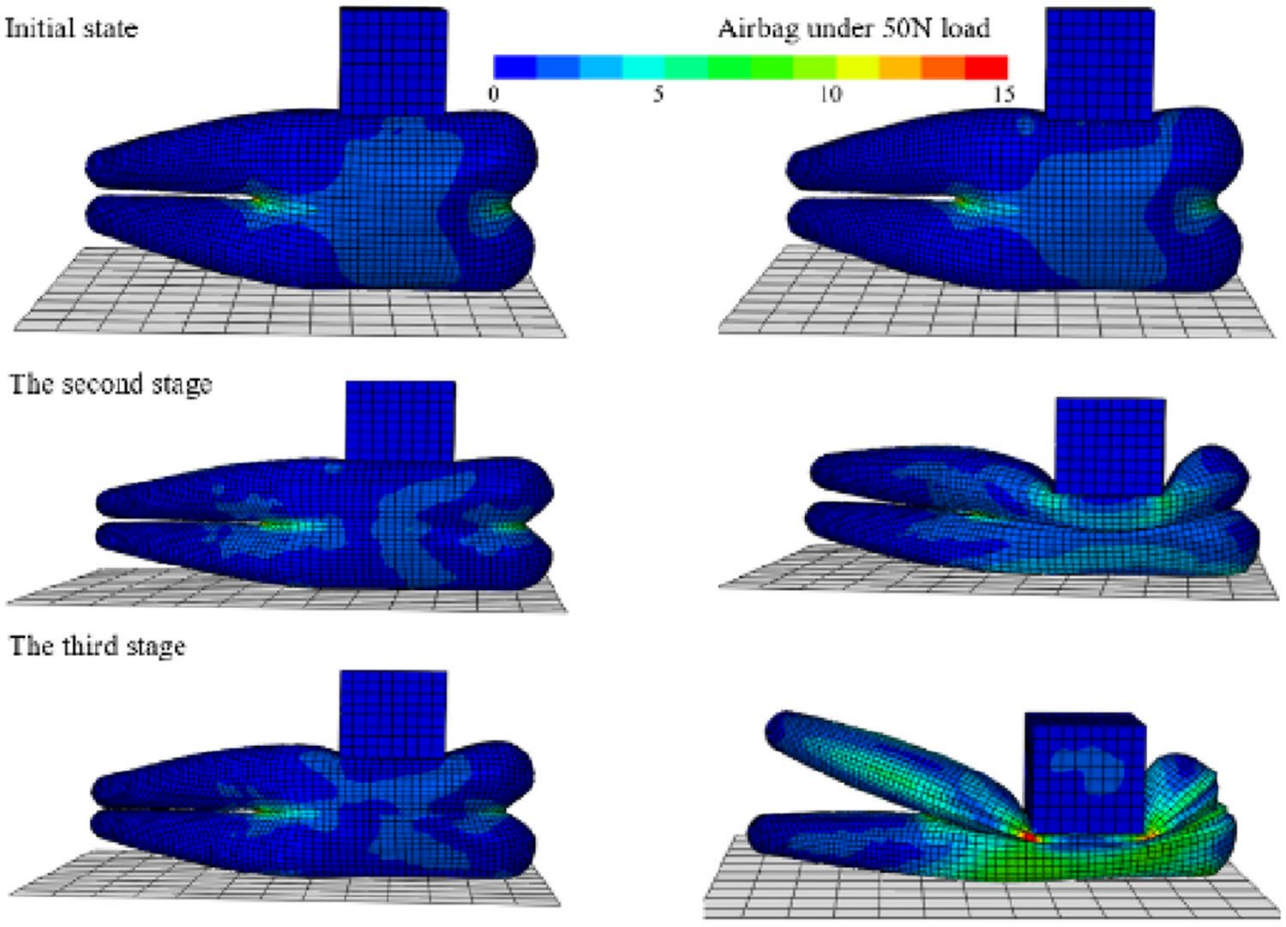
Finite element model of airbag loading process. Comparison of airbag before and after loading under three gas quality conditions of 3.5 g, 2.5 g and 1.5 g.

**Figure 8:**
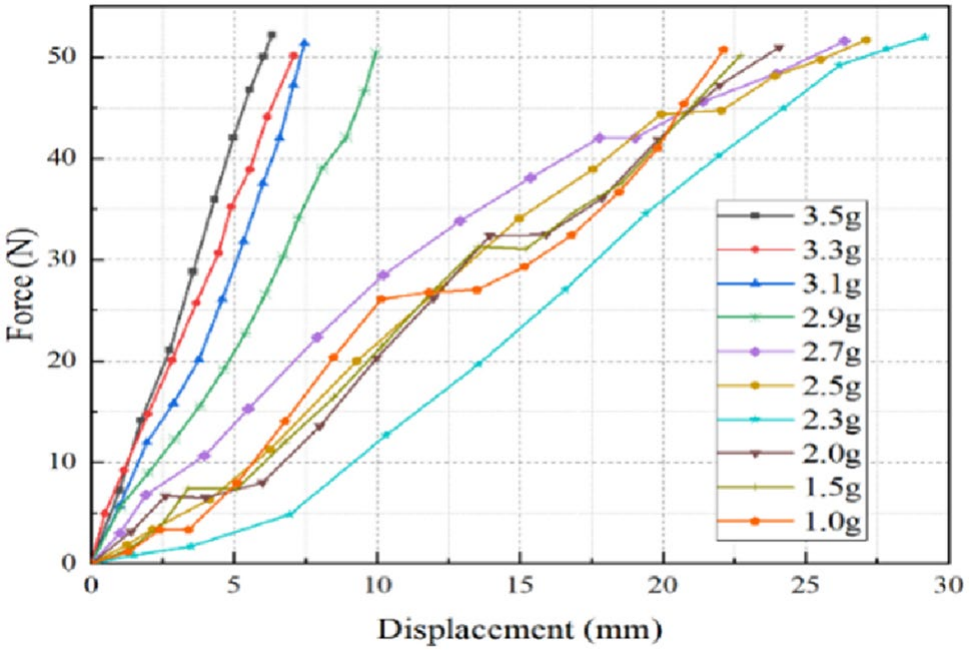
Correlation curve between airbag loading and displacement. Load–displacement simulation curves under different gas mass conditions.

In empirical terms, the pressure concentration position of a typical human body when lying down usually experiences a force range of 10 to 35 N^25^. Moreover, to ensure the mechanical parameter testing aligns with practical requirements, the external load applied to the airbag is scaled up by a factor of 1.5. Consequently, the experimental load conditions are set in the range of 0 to 50 N. The inflation process of the safety airbag involves controlled injection of air at a specific rate. Once full inflation is achieved, an external load block with a side length of 5 cm is applied at a loading rate of 10 mm/min (matching the loading rate of the universal material testing machine in the loading test). The loading time for each gas state is consistent with the test duration. Subsequently, after completion of the loading, the gas within the airbag is released equally, and the same external load is re-applied. As the loading block gradually exerts downward pressure, the contact surface of the wedge-shaped airbag with the loading block transitions from a curved surface to a flat surface equivalent in area to that of the loading block. Therefore, in this study, force data obtained from the highest node of the airbag before loading is utilized to characterize the contact force of the loaded airbag. Figure 7 depicts the deformation of the airbag at three distinct stages following a 50 N load, while Fig. 8 presents the load–displacement simulation outcomes under varying gas conditions within the airbag.

From Fig. 8, it can be found that the stiffness variation on airbag deflation process can be roughly divided into three stages. In the early stage (the gas mass in the airbag is more than 2.9 g in the figure), the gas mass in the airbag is relatively large. With the continuous loading, the corresponding compression amount increases as a linear function. The airbag stiffness remains constant during the loading process, in addition, as the airbag gradually deflates, its stiffness continues to decrease. As the airbag continues to deflate, the air bag stiffness variation characteristics enter the second stage (the gas mass in the airbag is from 2.7 to 2.3 g in the figure). At this stage, the airbag stiffness is no longer constant, and there is a compression stagnation phenomenon during the loading process. Same as the first stage, its stiffness decreases as the airbag gas mass continues to decrease. In the last stage of the deflation process (the gas mass in the airbag is from 1.5 to 1.0 g in the figure), the stiffness still exhibits the variable stiffness characteristics, but unlike the previous two stages, its stiffness increases as the gas mass decreases. The aforementioned findings are notably intriguing and hold significant implications for the precise control of airbag stiffness and personalized adjustments tailored to diverse users. In situations where the airbag material remains consistent and demonstrates a propensity to attain stability under loading conditions, coupled with an airbag cross-sectional shape that remains relatively uniform, the stiffness which a fundamental intrinsic attribute of the airbag in counteracting deformation exhibits minimal variation with alterations in external loading. Consequently, the simulation outcomes tend to manifest approximate linearity. Given the importance of this phenomenon, the ensuing elements of this paper will comprehensively delve into its intricate details and implications.

### Stiffness properties measuring experiment for a single airbag

**Figure 9:**
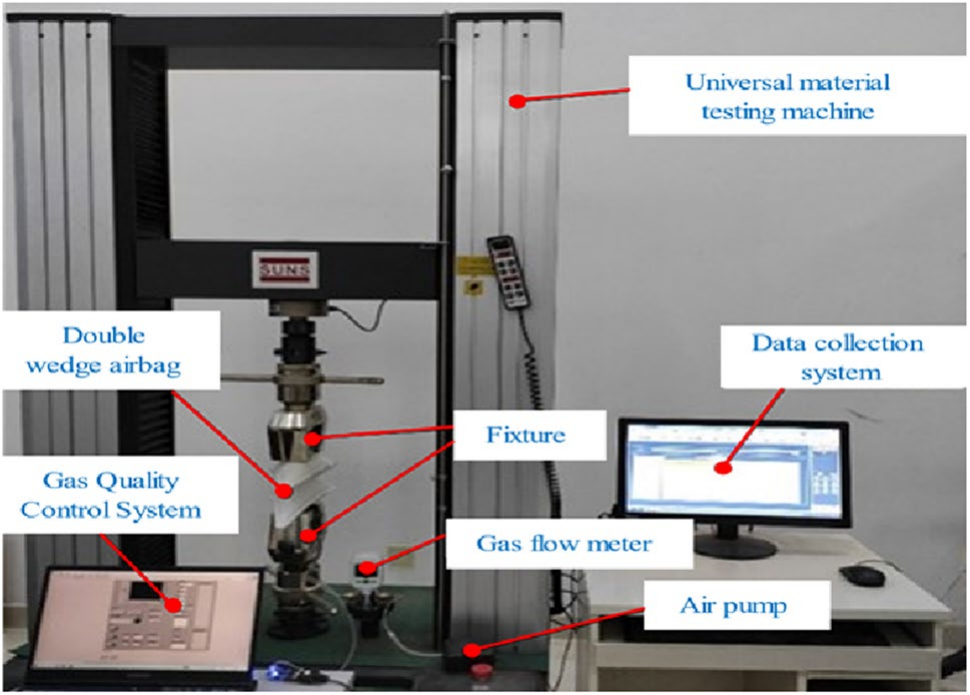
The single wedge-shaped airbag test platform. The universal material testing machine is used to load the airbag and record the experimental data, and the internal pressure acquisition module is used to read the airbag internal pressure data.

The airbag stiffness test platform is shown in Fig. 9. The components of the airbag test platform include a universal material testing machine, a data acquisition system, a double wedge-shaped airbag, an airbag control system, a gas mass flow meter, an air pump and a fixture. The air pump is the airbag air source, and the airbag control system realizes its inflation and deflation. The loading process is completed by the computer servo-controlled universal material test, which automatically collects and processes the load and displacement test data, and draws the test curve to ensure the control accuracy and measurement accuracy.

Before carrying out the airbag mechanical parameter test, the airbag gas mass and the range of the external load must be determined. The gas mass, that is, product of accumulated air flow and density in the airbag, were measured by gas mass flowmeter; the external load range is the same as consistent with the simulation above. The initial group was inflated until the airbag was fully filled, the gas mass was 3.5 g, the deflation volume of each group was consistent with the simulation above, and the minimum gas mass was 1.0 g. In addition, the independent variable of the airbag mechanical parameter test is the gas mass, and the dependent variables are the external loads, the airbag original height (the vertical height of the airbag under unload condition) and the displacement. Before each test, a vernier caliper is used to measure the wedge-shaped airbag original height on unload conditions.

## Results and discussion

### Loading experimental results analysis

**Figure 10:**
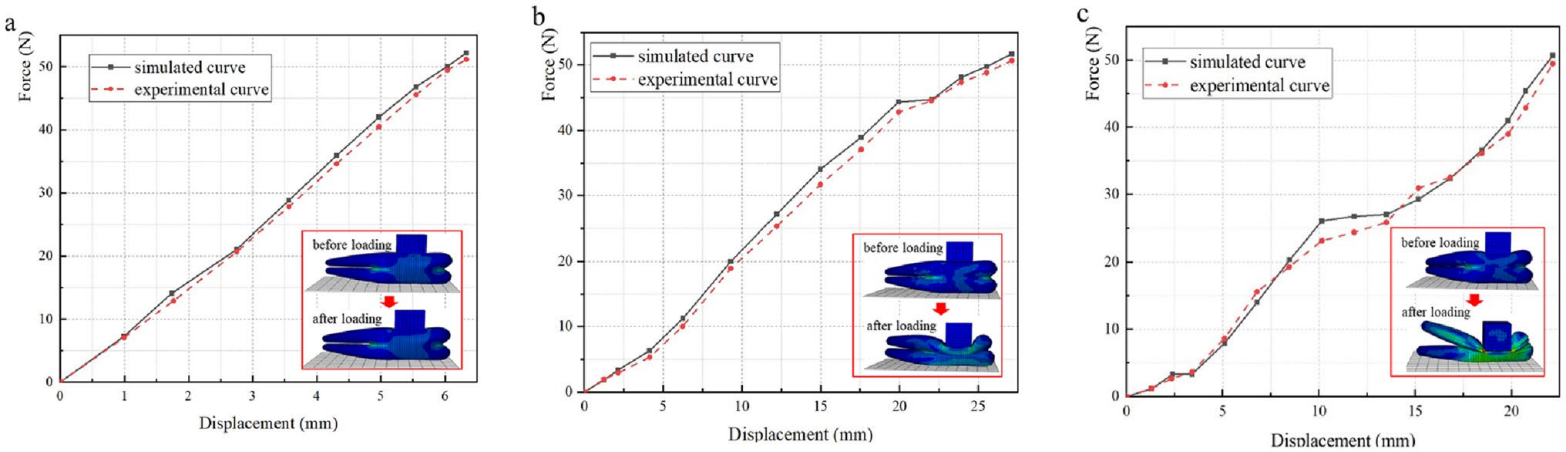
Comparisons of simulated curves and their corresponding experimental data in each stage under various airbag gas mass. (**a**) Phase 1. (**b**) Phase 2. (**c**) Phase 3.

The three stages airbag loading-compression comparison curves of different gas mass are shown in Fig. 10. Due to the limited length of the paper, the representative comparison curves of each stage are introduced in this paper (gas mass is 3.5 g, 2.5 g, 1.0 g), and the results of the comparison curves obtained under other gas mass conditions are consistent with conclusions above. It can be seen that, the experimental results are slightly lower than the simulation curves due to the errors and delays of the gas flowmeter and the pressure sensor, but the variation trend in the deflation process of these simulation curves is consistent with the actual experimental results, which proves the reliability of the simulation model. Thus, the modeling method is reliable to describe the airbag stiffness changes during the inflation and deflation of the airbag. This simulation modeling method can be effectively used into the design and development phases of nursing equipment airbags.

**Figure 11:**
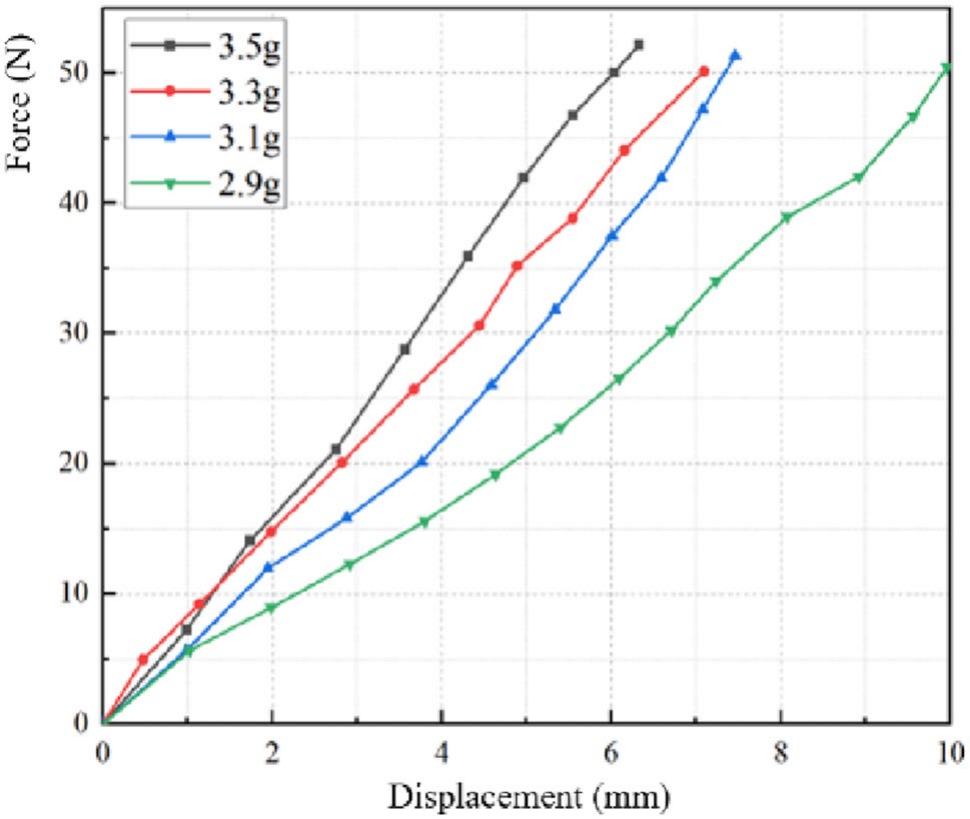
Simulated curves under various airbag gas mass (Phase 1). When the gas mass in the airbag is 2.9–3.5 g, the airbag deformation is small, and the load–displacement curve is a straight line.

From Fig. 11, it can be found that the airbag load–compression relationship is linear in a relatively saturated state, and the stiffness is constant. The main reason of phenomenon above is that the airbag has a certain volume, and the gas completely fills and supports which, so only vertical displacement occurs during the loading process, and which shape does not change much. In addition, since the input condition is the gas mass injected by the air pump, with the internal gas mass continuous reduction, the air molecules will be relatively loose, and the density and internal pressure will change accordingly. Therefore, the airbag stiffness variation rate in the first stage increases rapidly with the gas mass decreases.

**Figure 12:**
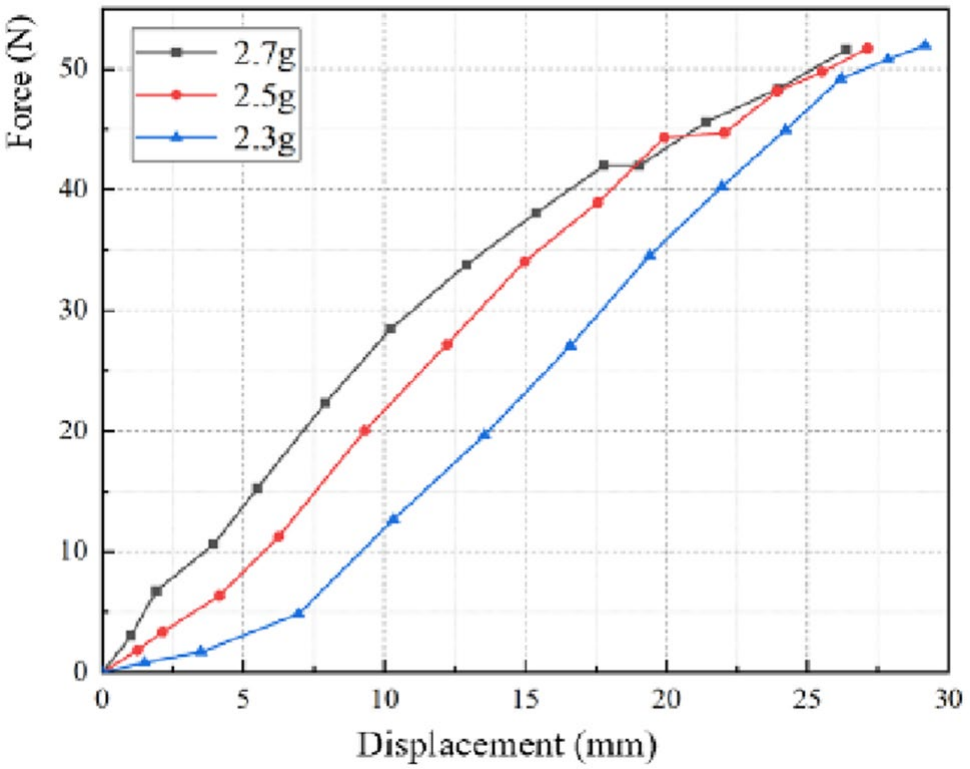
Simulated curves under various airbag gas mass (Phase 2). When the gas mass in the airbag is 2.3–2.7 g, the load–displacement curve is a curve, and the stiffness is no longer constant.

Figure 12 is a curve fitting graph of the second stage wedge-shaped airbag load–displacement curve. The load–displacement curve has a sudden stiffness variation at the beginning and end, but it is approximately a linear function in the main force range during the human body lying, and the variation rate of three curves (airbag stiffness) decreases as the gas mass decreases. The phenomenon above is mainly due to the insufficient inflation inside the airbag, which leads to the gas molecules free diffusion and movement. Therefore, during the loading process, the constant change of the airbag internal pressure leads to the gas mass and volume variation, and then the cross-sectional shape of the airbag changes accordingly, which finally leads to a sudden change of the airbag stiffness.

**Figure 13:**
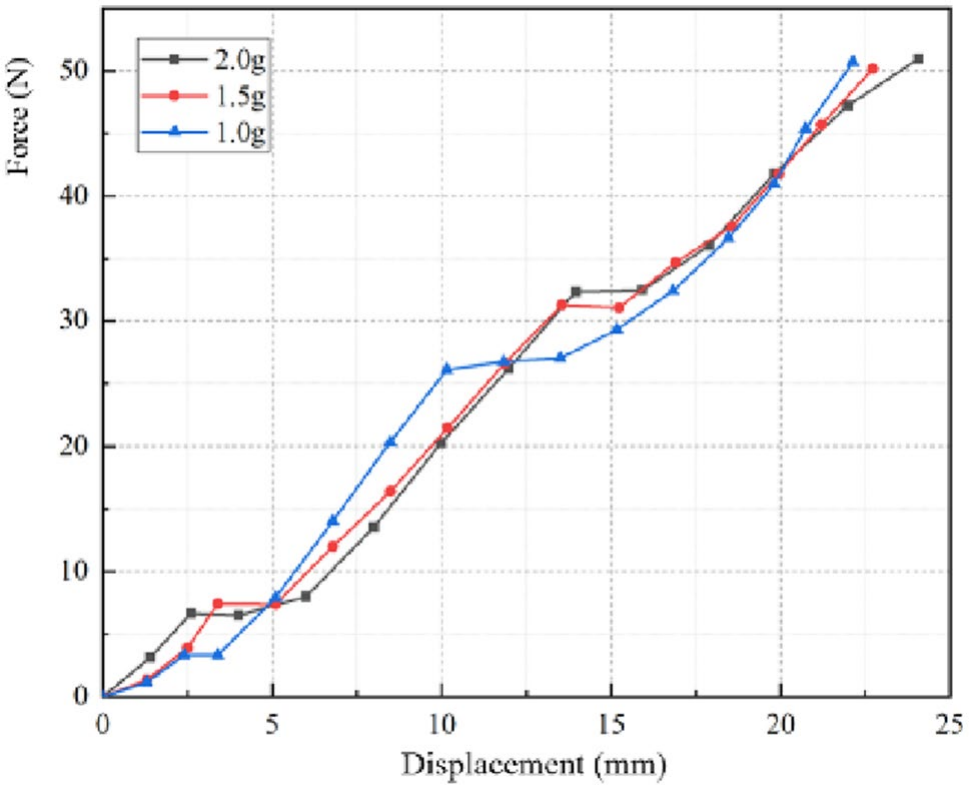
Simulated curves under various airbag gas mass (Phase 3). When the gas mass in the airbag is 1.0–2.0 g, the load–displacement curve is a curve, and the airbag stiffness gradually increases.

The load–displacement curve of the third stage is similar to that of the second stage, and its internal principle is basically the same, in the main force range, it can also be approximated as a constant stiffness. However, it can be seen from Fig. 13 that, unlike the phenomenon above, as the airbag gas mass continues to decrease, the airbag stiffness increases. After simulation analysis and observation of loading experiments, it is found that in the third stage, the airbag cannot be fully expanded after loading, and the gas is mainly concentrated in the lower airbag. Consequently, the reduced gas mass results in a smaller initial overall volume, which in turn results in an increased airbag stiffness.

### Mathematical relationship between the inflation height and internal pressure of nursing bed airbag

For the established airbag loading simulation models with various gas mass conditions, the relationship between the airbag load and its compression displacement can be studied separately. Furthermore, although there are various values of input conditions (such as airbag gas mass, airbag internal pressure, and external load) for airbag loading simulations, the correlation curves obtained from these simulations have similar shapes and trends. Furthermore, although there are various values of input conditions (such as airbag gas mass, airbag internal pressure, and external load) for airbag loading simulations, the correlation curves obtained from these simulations have similar shapes and trends. Therefore, it is of great significance to find the relationship between airbag stiffness and its inflation mass, and then exploring the comfort characteristic function of a given airbag to achieve accurate pressure dispersion of airbag nursing appliance for users with different physical characteristics.

Based on the conclusion, in the first stage, the airbag cross-sectional shape remains unchanged during the entire loading process, the load–displacement relationship of the wedge-shaped airbag is proportional, and the airbag stiffness is constant; in the second stage, at the beginning of loading, the cross-sectional shape changes abruptly, so the airbag loading-displacement relationship is an approximate multi-segment straight line, which the airbag stiffness can be treated as a constant stiffness in the main loading range; In the same way, in the final stage of deflation, due to the less gas in the bag, the shape of the air bag changes greatly, and the air bag stiffness presents a multi-stage phenomenon, but the stiffness is still constant stiffness in the force range above.

In this research, a significant training dataset is employed, and feature elimination was performed to exclude irrelevant or redundant attributes. This step resulted in the reduction of model complexity and the mitigation of overfitting risks. Moreover, diverse polynomial orders are explored throughout the fitting process to ascertain the optimal fitting model. This approach eliminates the need for an independent test set and effectively addresses overfitting concerns. Notably, this strategy not only streamlines the fitting procedure but also augments the simulation model’s overall capacity for generalization. Consequently, within the primary force range experienced during human body lying (10–35 N), a correlation curve is polynomial fitted using MATLAB, leading to the derivation of the subsequent parameter formulas (with a 95% confidence interval and R2 = 0.9982).

18 $$k={c}_{1}\times {x}^{5}+{c}_{2}\times {x}^{4}+{c}_{3}\times {x}^{3}+{c}_{4}\times {x}^{2}+{c}_{5}\times x+{c}_{6},$$

19 $${c}_{1}=-0.945 \left(-0.977,-0.913\right),$$

20 $${c}_{2}=8.394 \left(8.683, 8.114\right),$$

21 $${c}_{3}=- 25.707 \left(-26.594,-24.850\right),$$

22 $${c}_{4}=32.714 \left(31.623, 33.843\right),$$

23 $${c}_{5}=- 17.181 \left(-17.774,-16.608\right),$$

24 $${c}_{6}= 6.196 \left(5.989, 6.410\right),$$

where *x* is the gas mass (g), *k* is the airbag stiffness (N/mm), *c* is the fitting parameter.

According to the relationship above, the airbag stiffness properties is adjusted by controlling the total mass of air filled into the airbag, and then the airbag is inflated to a specific inflation volume (airbag stiffness) to avoid effective problems such as pressure sores or poor comfort, which greatly simplifies the personalized adaptation control of the multifunctional flexible airbag nursing equipment. By analyzing the simulation results above, it is not difficult to find that the variable airbag stiffness has defects in the quantitative control of the nursing airbag array unit. However, in the main force range, the airbag stiffness presents a fixed stiffness characteristic, so the airbag stiffness model can accurately predict the stiffness of the airbag array unit above, which provides a theoretical basis for the individual adaptation of airbag apparatus.

## Conclusion

The majority of existing modeling analyses pertaining to airbags primarily focus on industrial applications and often lack qualitative assessments of airbag stiffness characteristics. The primary innovation of this paper lies in the comprehensive analysis of the stiffness attributes of wedge-shaped airbags, approached through both experimental and simulation perspectives, and the subsequent establishment of a dedicated numerical simulation model. To achieve precise and flexible control, as well as personalized adaptation of an array airbag nursing apparatus, this paper delves into the numerical modeling methodology of the double wedge-shaped airbag loading process with varying inflation volumes. In the pursuit of this objective, several key steps are undertaken. Initially, the hyperelastic material constitutive model is established based on the uniaxial tensile experimentation conducted on the airbag film materials. This model serves as the foundation, supplying essential material property parameters necessary for the subsequent modeling of the airbag loading process, encompassing diverse gas masses. Subsequently, the loading process of the airbag is meticulously simulated, culminating in the formulation of a mathematical relationship between airbag stiffness and its corresponding internal gas mass. In summary, the key findings of this study are outlined below:

1. Based on the validation through airbag loading tests, the simulation model established in this paper demonstrates a favorable level of fitting accuracy. As a result, the modeling approach proposed herein holds the potential to offer valuable insights and guidance for the simulation modeling and stiffness characterization of hyperelastic material properties. Building upon the aforementioned model, the relationship between load and airbag compression under varying gas masses is derived. Subsequently, the mathematical correlation between the stiffness of the double wedge-shaped airbag and its internal inflation volumes is elucidated. This relationship holds the promise of precise control over the airbag array unit and serves as a foundational framework for implementing a differentiated and intelligent control strategy for the airbag nursing device. Such a strategy could effectively distribute pressure and mitigate the risk of pressure ulcers for users.
2. The presented method can be utilized into the design and optimization of airbag array unit, which can be used for airbag nursing mattresses or cushions. In addition, the simulation results and the manifestations presented in this paper are of great significance for the optimization of the airbag structure, especially the airbag shape (wedge diagonal angle and cross-sectional shape).

Currently, research on the stiffness characteristics of airbags primarily focuses on industrial applications, lacking suitable load simulation models for airbags used in medical care mattresses. As a result, accurate assessment of the stiffness characteristics of airbag mattresses is challenging, preventing the realization of personalized adjustment and quantitative control for pressure-relief air mattresses. Addressing the aforementioned issues, this paper establishes the simulation model for the loading process of a wedge-shaped single airbag and elucidates the variations in its stiffness characteristics. However, the simulation model above does not account for the coupling of multiple airbags, the overall stiffness characteristics of airbag arrays, and the impact of airbag coupling on the stiffness of individual wedge-shaped airbags, which necessitates further investigation. Hence, building upon the foundation of the single airbag loading simulation model, the future research direction of this study involves developing a simulation model for the loading of an airbag array with multiple coupled airbags, representing real-world usage scenarios. This will facilitate the exploration of the overall stiffness characteristics of airbag arrays and the influence of airbag coupling on the stiffness of individual wedge-shaped airbags.

## Data Availability

The data used to support the findings of this study are included within the article.
